# Home-Based Virtual Reality Training for Enhanced Balance, Strength, and Mobility Among Older Adults With Frailty: Systematic Review and Meta-Analysis

**DOI:** 10.2196/67146

**Published:** 2025-07-18

**Authors:** Hammad Alhasan, Elaf Alandijani, Lara Bahamdan, Ghofran Khudary, Yara Aburaya, Abdulaziz Awali, Mansour Abdullah Alshehri

**Affiliations:** 1 Department of Medical Rehabilitation Sciences, Faculty of Applied Medical Sciences, Umm Al-Qura University, Makkah 24382, Saudi Arabia, 966 555516226

**Keywords:** older adults with frailty, balance, strength, functional mobility, home-based training, virtual reality, exergames, systematic review, older adults, PRISMA

## Abstract

**Background:**

Frailty is a geriatric syndrome associated with increased risk of falls, hospitalization, and reduced quality of life. Traditional exercises may be unsuitable for older adults with frailty due to mobility issues and accessibility barriers. Virtual reality (VR) offers an engaging, home-based alternative by providing interactive training with real-time feedback. VR interventions have shown potential benefits for improving balance, strength, and mobility.

**Objective:**

This systematic review and meta-analysis aimed to evaluate the effectiveness of VR-based home training programs in improving balance, strength, and mobility among older adults with frailty and prefrailty.

**Methods:**

A systematic search was conducted in PubMed, Scopus, and Web of Science from inception to November 1, 2023, using terms related to older adults, frailty, virtual reality, balance, mobility, and strength. Eligible studies included randomized and nonrandomized trials involving adults with frailty or prefrailty aged ≥65 years who received home-based VR interventions aimed at improving balance, strength, or functional mobility. Comparator groups included no intervention, traditional exercise, or standard care. Studies involving participants with neurological or cognitive disorders were excluded. Study quality was assessed using the Physiotherapy Evidence Database scale. A random-effects meta-analysis was performed to calculate pooled mean differences (MD) and 95% CIs for 3 primary outcomes: Berg Balance Scale, Timed Up and Go, and Chair Stand.

**Results:**

A total of 1063 records were identified, with 1023 screened after duplicate removal. Six studies met the inclusion criteria, involving 407 participants (mean age 75.2, SD 6.4 y), of whom 198 were allocated to VR interventions and 159 to control groups. VR interventions lasted a mean of 13.3 (SD 7.7) weeks, with an average of 39.6 (SD 5.2) sessions lasting 25.3 (SD 5) minutes. Methodological quality was high in 5 studies (mean Physiotherapy Evidence Database score=5.6, SD 1.3). Four studies were included in the meta-analysis. Significant improvements were observed in balance, as measured by the Berg Balance Scale (MD=3.62; 95% CI 2.29‐4.95; *P*<.001; *I*²=0%). No significant effects were found for mobility (Timed Up and Go: MD=−0.37; 95% CI −1.16 to 0.41; *P*=.35; *I*²=0%) or strength (Chair Stand: MD=−0.20; 95% CI −1.70 to 1.29; *P*=.79; *I*²=21%).

**Conclusions:**

VR-based home exercise interventions show promise in improving balance among older adults with frailty and prefrailty. However, their effects on strength and functional mobility remain unclear. Variability in study designs and outcome measures limits the generalizability of current findings. Further high-quality research is needed to determine optimal VR training protocols and assess long-term adherence and clinical effectiveness.

## Introduction

Frailty is a major concern for older adults, significantly affecting their well-being and quality of life [1,2]. It is characterized by a significant decline in the performance of various physiological systems and lacks a universal phenotype, signifying its heterogeneity as a geriatric syndrome [3,4]. Instead, it varies among individuals, considering their unique characteristics and circumstances, with a consensus that frailty is characterized by an increased vulnerability to adverse health outcomes [5,6]. Individuals with frailty, who are prone to experiencing functional decline and disability, face a higher risk of falls, hospitalization, and mortality [7,8]. Therefore, falls are a major concern for older adults with frailty, as they can lead to loss of autonomy, injuries, and even death [9,10].

Frailty can be categorized into 3 stages: prefrailty, frailty, and frailty complications. In the prefrailty stage, individuals may experience 1 or 2 symptoms that directly indicate limitations in their physical function or health; with early intervention and appropriate responses, successful management of these challenges is possible [11]. The frailty stage is characterized by hallmark symptoms such as weight loss, exhaustion, low physical activity, slowness, and weakness that lead to limitations in the individual’s functioning and worsening of the overall quality of life [12]. The frailty complications stage occurs when an individual’s functional independence is significantly impaired with accompanying behavioral patterns that may lead to death [13]. The Fried Frailty Phenotype stands out as a widely used tool for assessing frailty. It assesses physical frailty using 5 criteria: unintentional weight loss, low energy or self-reported exhaustion, reduced grip strength, reduced physical activity, and slowness by slowed walking speed. When 1 or 2 criteria are present, the individual is considered to be in a prefrail state, while the presence of more than 2 criteria indicates frailty [14,15].

Frailty prevalence increases with age, affecting 46% of older adults in the prefrail stage and 15%‐11% in the frail stage [16]. Socioeconomic factors, nutritional status, and ethnic background also play significant roles in frailty prevalence. Longitudinal studies on frailty progression are limited, but some indicate that frailty status can improve, remain stable, or worsen over time [17,18]. These statistics underscore the widespread impact of frailty among older adults, highlighting the need for targeted interventions.

Given the above, society is faced with the challenge of finding effective rehabilitation solutions to promote healthy aging [19,20]. Traditional exercises are often not preferred by older adults due to factors such as lack of motivation, perceived physical limitations, and the repetitive and monotonous nature of the exercises [21-23]. Virtual reality (VR) technology presents a promising alternative that could effectively address these challenges as it provides practical and easy-to-use solutions [24-28]. In this review, VR refers to interactive, digital systems that simulate task-oriented environments to encourage physical rehabilitation, which includes platforms such as sensor-enabled gaming platforms, exergames, and nonimmersive VR [29,30]. This definition incorporates motion-tracking systems that do not require the use of head-mounted displays but require real-time feedback and user engagement through physical movement, which are commonly used in VR-based rehabilitation interventions [31].

In comparison with traditional exercises, VR offers numerous advantages, such as structured guidance, real-time feedback, and adaptable difficulty, enabling users to engage safely within their abilities, especially for those at risk of falling [32]. Additionally, the interactive elements of VR have been shown to increase motivation, adherence, and cognitive engagement [26,33-35]. It should also be emphasized that VR-based exercises can significantly improve motor and cognitive functions [29,32,36,37]. A recent randomized controlled trial (RCT) compared the effect of VR training to Otago exercises [38]. Balance was used as an outcome, and the results indicated that the VR group showed significant improvements compared to the Otago exercise group. However, the study used a pre-post intervention design without a control group, which makes it challenging to attribute improvements solely to the VR intervention. Additionally, the study’s findings may not apply to older adults with frailty as they recruited community-dwelling older adults. Similarly, a trial compared the effect of traditional versus VR treadmill on mobility and cognition among individuals with frailty [39]. Both modalities yield positive effects, but there is a preference for VR over traditional treadmill exercises due to the added benefit of cognitive improvement. A recent systematic review found that supervised VR training in rehabilitation settings can improve balance and reduce fall risk among older adults with frailty [40,41].

While traditional, center-based training has demonstrated the aforementioned benefits, older adults with frailty often face challenges to participating in traditional center-based exercise due to mobility limitations, fear of falling, and transportation challenges [42,43]. The COVID-19 pandemic further highlighted the importance of remote care models for this population [44,45]. These limitations emphasize the need for other approaches that are both safe and accessible. VR-based training may be specifically suitable for older adults with frailty, who frequently face challenges to engaging in traditional exercise due to impaired mobility or fear of falling [36,46]. Hence, home-based VR training is increasingly being studied as a feasible method to enhance physical activity among individuals with frailty. This prompts the need to investigate whether similar findings can be achieved through the use of VR at home, taking into consideration its ease of access, affordability, and other advantages. Therefore, the objective of the current systematic review was to examine the effectiveness of VR as home-based training on balance, strength, and mobility outcomes among older adults with frailty or prefrailty.

## Methods

### Protocols and Registration

The current systematic review was registered on PROSPERO with the following registration (CRD42023478330 [47]).

### Data Sources

The PRISMA (Preferred Reporting Items for Systematic Reviews and Meta-Analysis) guidelines were followed for this review. The PRISMA item checklist can be found in Checklist 1. The search time frame was from inception to November 1, 2023. The goal was to identify recent studies on the effects of VR training for enhanced balance, strength, and mobility at home among older adults with frailty and prefrailty. Two authors independently performed searches in the following databases: Scopus, Web of Science, and PubMed.

### Search Strategies

The search terms were specific to each database. The following is an example of the search terms used in Scopus: risk of fall OR balance OR strength OR function AND frail OR prefrail AND older adult AND virtual reality OR video games OR mobile game. A detailed overview of the search terms and strategies used is provided in Multimedia Appendix 1.

### Selection Criteria

The study comprised all English-language papers, including those that used a single-group design in which a VR as a home-based exercise intervention was compared with no intervention or other interventions for enhanced strength, balance, and mobility among older adults with frailty or prefrailty. The PICOS (Population, Intervention, Comparison, Outcome, and Study Design) framework for the current review was as follows:

- Of population (P), older adults with frailty or prefrailty aged 65 years or more.
- Of intervention (I) VR, a home-based exercise that is used to improve balance, strength, and mobility.
- Of comparison (C) no intervention, traditional exercises, or standard care.
- Of outcomes (O) balance, strength, and mobility measured using validated outcome measures.
- Of study design (S), RCT and non-RCT.

Table 1 summarizes the inclusion and exclusion criteria structured according to the PICOS framework.

**Table 1.** Summary of the inclusion and exclusion criteria.

Category	Inclusion criteria	Exclusion criteria
Population	Older adults with frailty or prefrailty (aged 65+ years)	Adults without frailty and younger populations (<64 years)
Intervention	VR^a^, video games, or mobile game-based interventions targeting balance, strength, or function	Interventions not involving VR or gaming, or not targeting balance, strength, or function
Comparison	Any (eg, standard care, other exercise modalities, or no intervention)	None required
Outcome	Outcomes related to balance, strength, and function	Studies not reporting functional outcomes related to balance, strength, mobility, or fall risk
Study design	Randomized controlled trials or clinical trials	Observational studies, reviews, or case reports
Language	English	Non-English publications
Publication type	Peer-reviewed full-text papers	Abstracts, dissertations, or protocols

a VR: virtual reality.

### Participants

The included studies comprised male or female older adults with a mean age of 65 years or older, described as older adults with frailty, older adults with prefrailty, aged, geriatric, or older adults living in the community, independently, in retirement centers or nursing homes. Studies that included participants with specific medical conditions, such as stroke, Parkinson disease, or cognitive impairment, were excluded. Table 2 summarizes the frailty status of participants across the included studies.

**Table 2.** Frailty status of participants in included studies.

Study	Frailty status (N)	Frailty status description
[48]	Frail (61)	Participants were explicitly described as frail, with reduced mobility.
[49]	Prefrail (202)	Participants were described as having a moderate fall risk.
[50]	Prefrail (59)	Participants showed fall risk indicators and used assistive devices.
[51]	Prefrail (30)	Participants were sedentary, nonexercising older adults with some support needs.
[52]	Frail (18)	All participants had a history of falls and lived in a nursing home.
[53]	Prefrail (37)	All participants were explicitly classified as prefrail.

### Interventions

The included studies explored various interventions to improve balance, mobility, and physical function; these included video-guided exercises with resistance bands, VR balance training guided by a physiotherapist, or sensor-based exergames exercised at home. Each intervention was compared to traditional exercise programs or participants’ usual activities to assess their effectiveness.

### Outcome Measures

The included studies assessed the effectiveness of various VR interventions for older adults by measuring changes in physical function and mobility. Outcomes such as the knee extension strength test and Sit-to-Stand test were used to assess lower extremity strength, the Berg Balance Scale (BBS) test was used to assess balance, and the Timed Up and Go (TUG) test was used to assess functional mobility.

### Quality Assessment

The Physiotherapy Evidence Database (PEDro) assessment tool was used to assess the methodological quality of the included studies [54]. The total PEDro score reflected the quality of the study as follows: a total score of ≥6 indicated high quality, 4‐5 represented fair quality, and ≤3 indicated poor quality [55].

### Data Extraction

The reviewers independently assessed the trials for eligibility by reviewing the titles and abstracts. If a paper title or abstract was deemed relevant, the full text was retrieved for evaluation against the inclusion and exclusion criteria. Any disagreement between the authors was resolved by the lead author. A data extraction form was created, and the data were extracted by the independent reviewers.

### Data Analysis

A random-effects meta-analysis was performed using Review Manager (version 5.4; Cochrane) software. Three primary outcomes were included: BBS, TUG, and Chair Stand (CS) tests. The purpose was to identify the mean difference (MD) in balance, risk of falls, and strength between VR groups and conventional intervention or control groups, and also to determine the overall treatment effect size. Heterogeneity was assessed with the *I*^2^ index, which has 4 classification levels: unimportant heterogeneity (0%‐40%), moderate heterogeneity (30%‐60%), substantial heterogeneity (50%‐90%), and considerable heterogeneity (75%‐100%) [56]. Effect sizes were calculated for all studies using Cohen *d*, with <0.2 indicating a “trivial” effect size, with 0.2 indicating a “small” effect size, 0.5 indicating a “medium” effect size, and 0.8 indicating a “large” effect size [57].

## Results

### Overview

**Figure 1.**
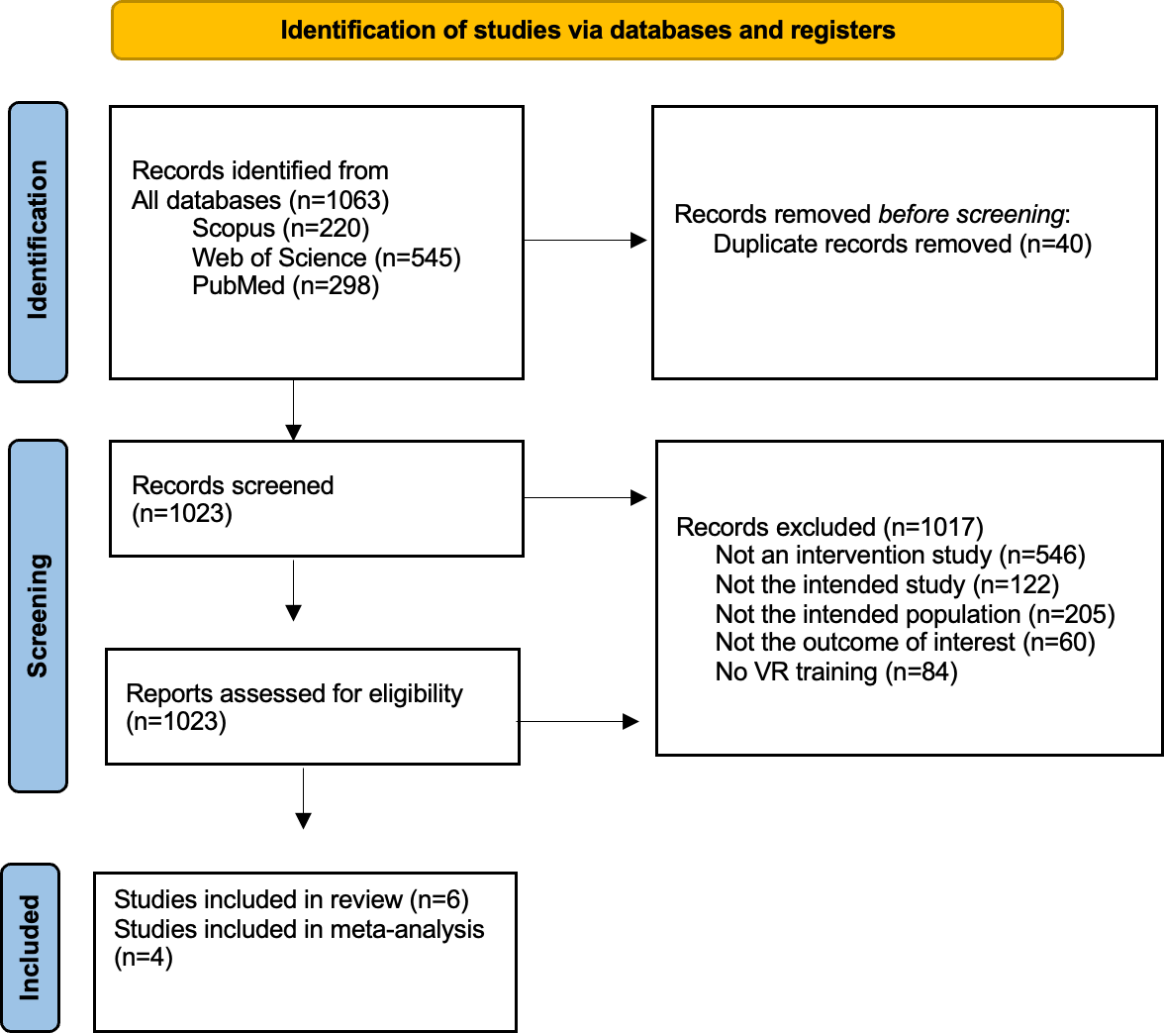
The results of the literature search conducted on November 1, 2023. VR: virtual reality.

A total of 1063 papers were identified as relevant; 40 were duplicates. The remaining 1023 papers were screened. After the initial screening, 1017 papers were excluded based on the titles and abstracts. The final review included 6 papers. The selection process for this systematic review is presented in the flow diagram in Figure 1.

### Methodological Quality

The mean PEDro score was 5.6 (SD 1.3) with 5 studies [48,50-53] graded as high quality and 1 [49] as poor quality. Table 3 presents the results of the quality assessment of the included studies.

**Table 3.** Physiotherapy evidence database scale assessment for included studies.

Study	1^a^	2^b^	3^c^	4^d^	5^e^	6^f^	7^g^	8^h^	9^i^	10^j^	11^k^	Total
[48]	✓^l^	✓	N^m^	✓	N	N	N	✓	✓	✓	✓	6/10
[49]	✓	N	N	N	N	N	N	N	✓	✓	✓	3/10
[50]	✓	✓	N	N	N	N	✓	✓	✓	✓	✓	6/10
[51]	✓	✓	N	✓	N	N	N	✓	✓	✓	✓	6/10
[52]	✓	✓	N	✓	N	N	✓	✓	N	✓	✓	6/10
[53]	✓	✓	✓	✓	N	N	✓	✓	N	✓	✓	7/10
Total	6/6	5/6	1/6	4/6	0/6	0/6	3/6	5/6	4/6	6/6	6/6	

a 1: Eligibility criteria.

b 2: Random allocation.

c 3: Concealed allocation.

d 4: Baseline comparability.

e 5: Blind people.

f 6: Blind therapists.

g 7: Blind assessors.

h 8: Adequate follow-up.

i 9: Intention-to-treat analysis.

j 10: Between-group comparisons.

k 11: Point estimates and variability.

l ✓: yes.

m N: no.

### Characteristics of Included Studies

#### Participants and Study Designs

Four of the included studies [48,49,51,52] were RCTs and the remainder were experimental non-RCTs [50,53]. The total number of participants recruited studies was 407, with a mean age of 75.2 (SD 6.4) years, while 357 participants were included in the analysis. Of those analyzed, the VR groups comprised 198 participants, and the control groups comprised 159 participants. The number of individuals in the VR groups ranged from 7 to 63, with a mean of 33 (SD 21.1). In the control groups, the number of individuals ranged from 11 to 61, with a mean of 31.8 (SD 20.1). All studies were conducted at home or in nursing homes and in public home care. Characteristics of included studies are summarized in Table 4.

**Table 4.** Characteristics of included studies.

Study	Design	Total number of participants	Intervention	Training type for the intervention group	Total sessions, weeks, duration	Number of samples analyzed	Outcome measures	Effect size
[48]	RCT^a^	61	IG^b^: video + resistance band; CG^c^: standard care	Individual home-based exercise using video and booklet	5 months, 3 sessions/week, 26 min/session	IG=25; CG=28	Chair Stand	d=0.32
[49]	RCT	37	IG: step pad + CSRT^d^; CG: usual activities	Home-based interactive step game	8 weeks, 2‐3 sessions/week, 15‐20 min/session	IG=15; CG=17	TUG^e^, Chair Stand, proprioception	TUG: d=0.14; CS: d=0.29
[50]	Pilot study	148	IG1: Microsoft-Kinect exergames; IG2: SMT^f^; CG: usual activities	Unsupervised home programs (WEBB^g^, Otago, SMT)	16 weeks, 3 sessions/week	IG1=24; IG2=39; CG=61	TUG, Chair Stand, proprioception	TUG: d=0.24; CS: d=0.16
[51]	RCT	100	IG: Kinect video games; CG: balance, stretching, or strength	Play Kinect games supervised by nurse	6 weeks, 5 sessions/week, 30 min/session	IG=48; CG=42	TUG, BBS^h^	BBS: d=1.10; TUG: d=0.05
[52]	RCT	21	IG: balance training with BTS NIRVANA^i^ VR^j^; CG: conventional balance exercises	VR-based balance exercises supervised by a PT^k^ in a nursing home	6 weeks, 3 sessions/week, ~35‐45 min/session	IG=7; CG=11	TUG, BBS	TUG: *P*=.01; BBS: d=0.71
[53]	Pilot study	40	IG: Otago-based exercise; CG: none	Lower-body strength and balance exercises	6 months (3 supervised + 3 unsupervised)	40	TUG, Chair Stand	TUG: *P*=.006; CS: *P*=.03

a RCT: randomized control trial.

b IG: intervention group.

c CG: control group.

d CSRT: choice stepping reaction time.

e TUG: Timed Up and Go.

f SMT: Step Mat Training.

g WEBB: Weight-Bearing Exercise for Better Balance.

h BBS: Berg Balance Scale.

i BTS NIRVANA: innovative therapeutic systems aiding the rehabilitation process of patients affected by neuro-motor disease by multisensorial stimulation.

j VR: virtual reality.

k PT: physical therapist.

#### Number and Duration of Intervention

The duration of VR sessions ranged from 6 to 24 weeks, with a mean of 13.3 (SD 7.7) weeks. The total number of sessions ranged from 16 to 120, with a mean of 39.6 (SD 5.2) sessions. The length of the sessions was 10 to 50 minutes, with a mean of 25.3 (SD 5) minutes. Descriptions of the exergame interventions used in the included studies can be found in Table 5 and Multimedia Appendix 2.

**Table 5.** Descriptions of VR^a^ or exergame interventions used in included studies.

Study	VR tool	Description
[48]	Television-based video exercise program	Participants followed a prerecorded exercise video featuring strength and balance activities based on the Otago Exercise Program.
[49]	Dance Dance Revolution style step pad game	Participants used a step pad linked to a screen to play rhythm-based games, requiring directional steps and incorporating cognitive challenges to enhance executive function.
[50]	Microsoft Kinect-based exergame	Participants engaged in movement-based games that require stepping, shifting, and reaching, using motion-sensor technology.
[51]	Microsoft Kinect-based exergame	Participants used a motion-sensing camera system to perform stepping, squatting, and weight-shifting, guided by visual cues on a screen.
[52]	Nintendo Wii Fit system	Participants performed exergames using the Wii Balance Board, performing static and dynamic activities with real-time visual feedback.
[53]	Tablet-based video program with wearable motion sensors	Participants used a tablet app that delivered exercise videos inspired by the Otago program, and a necklace-worn motion sensor was used for activity monitoring and feedback.

a VR: virtual reality.

### Qualitative Analysis

#### BBS

Two studies [51,52] used the BBS as a key outcome measure. Significant improvements were observed in VR groups. Both studies assessed BBS postintervention within 6 weeks.

#### TUG

Five studies [49-53] used the TUG as a key outcome measure. Significant improvements were found in the VR group in 3 studies [50-52], where 2 studies [51,52] assessed TUG postintervention within 6 weeks and 1 within 6 months of follow-up [50]. The remaining 2 studies [49,53] did not show significant improvements for all groups, where 1 study [49] assessed TUG postintervention within 8 weeks and the other one [53] within 16 weeks.

#### Strength

Two studies [49,50] used knee extension strength as a key outcome. Both studies did not show significant improvements in any group. Four studies assessed CS performance as a key outcome measure [48-50,53]. All studies did not show significant improvements for all groups.

### Meta-Analysis

#### Overview

**Figure 2.**
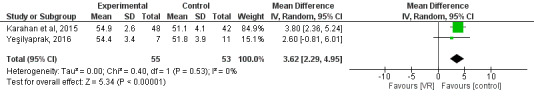
Forest plot for the mean difference of the effect of VR compared with conventional interventions and control on the BBS; lower BBS mean score indicates higher risk of falling [51,52]. BBS: Berg Balance Scale; VR: virtual reality.

**Figure 3.**
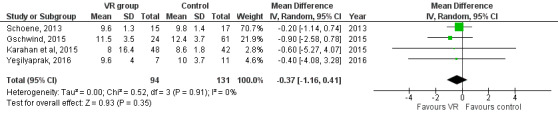
Forest plot for the mean difference of the effect of VR compared with conventional interventions and control on the time (in seconds) of the TUG; lower TUG mean score indicates better mobility performance [49-52]. TUG: Timed Up and Go; VR: virtual reality.

**Figure 4.**
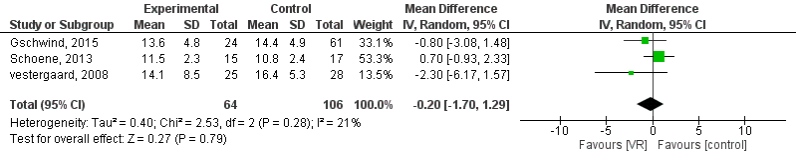
Forest plot for the mean difference of the effect of VR compared with conventional interventions and control on the Chair Stand test; lower mean score indicates better mobility performance [48-50]. VR: virtual reality.

Only four studies [49-52] were included in the meta-analysis. The study by Vestergaard et al [48] did not use standardized outcomes compatible for pooling, and the study byGeraedts et al [53] was a single-arm study without a control group, preventing calculation of comparative effect sizes. Therefore, both were excluded from the meta-analysis [43-46]. Figures 2-4 show the overall treatment effect size and the results of each study on the BBS, TUG, and CS.

#### BBS

Two [51,52] studies with 55 participants were eligible for inclusion in this meta-analysis. A forest plot revealed that VR yielded better results than conventional interventions and no intervention (control) in terms of improvements in postural control (MD=3.62; 95% CI 2.29 to 4.95; *P*<.001; *I*^2^=0%). For conventional interventions and the control, the results of the meta-analysis showed a lower mean score on the BBS, indicating a higher risk of falling (Figure 2).

#### TUG

Four studies [49-52] with 94 participants were eligible for inclusion in this meta-analysis. The forest plot showed no significant differences between the interactive video games and other interventions or the control on TUG (MD=−0.37; 95% CI −1.16 to 0.41; *P*=.35; *I*^2^=0%; Figure 3).

#### Strength

Three studies [48-50] with 64 participants were eligible for inclusion in this meta-analysis. The forest plot showed no significant differences between interactive video games and other interventions or the control on CS (MD=−0.20; 95% CI −1.70 to 1.29; *P*=.79; *I*^2^=21%; Figure 4).

## Discussion

### Principal Findings

This current systematic review aimed to identify studies that have investigated the use of VR training at home to improve balance, strength, and mobility among older adults with frailty and prefrailty. Although few studies have focused on this aim, the findings showed that VR was effective at improving balance but not strength and mobility. One potential reason for this could be due to limited experience with independent technology use among older adults and therapists, a challenge that has been validated in previous studies [41,58]. Nevertheless, the studies included in this review are examples of how technological advancements can reshape health care delivery and demonstrate that certain VR interventions can be used safely with older adults, as no study reported any safety hazards.

### Comparison to Prior Work

The findings from this review showed that home-based VR intervention produces great variability in effectiveness on different outcomes. On balance, both [51,52] found a significant effect on balance in the VR group when using the BBS. This finding aligns with a recent review [59], which also found a significant improvement in BBS scores following VR intervention in older adults with balance impairments. Similarly, another study [60] found that VR intervention significantly improved BBS scores in older adults residing in nursing homes compared with those living in communities. The populations studied in these studies were similar, indicating that these findings may be generalizable.

For strength outcomes, the CS test did not show significant improvements in 4 of the included studies. In the study by Vestergaard et al [48], the lack of improvement may be due to insufficient progression in the exercises included in the VR training group. Without adequate progression or intensity, participants may not have experienced meaningful gains in strength or functional mobility. However, this remains a possible explanation rather than a definitive conclusion. In the study by Gschwind et al [50], although the Step Mat Training (SMT) group demonstrated significant improvements in sit-to-stand times compared to the control group, no significant differences were found between the 2 intervention groups or between the intervention group and the control group. This limited improvement could suggest that Kinect training may not have been as effective in enhancing strength and balance. The intensity and specificity of exercises delivered through VR interventions are likely contributing factors. While some studies may have used tailored and progressively challenging tasks to enhance training efficacy, others may have implemented more generic routines that did not adequately target the specific mobility deficits of participants.

In contrast to mobility outcomes, home-based VR training’s effect on the TUG showed variation in the included studies. The lack of significant improvement could be attributed to the small sample size [49,50], which limited the ability to detect changes. This variation in effect on TUG could also be due to the specific design and characteristics of the VR interventions implemented in these studies. For instance, 1 study [49] used short-duration interventions of 10‐20 minutes per session, while another [51] used longer-duration interventions of 30 minutes per session. Similarly, the VR effect on knee extension strength did not show significant improvements in the 2 studies [49,50]. In the study by Schoene et al [49], the intervention did not specifically target knee extension strength or range of motion, focusing more on stepping performance and cognitive parameters related to fall risk. In contrast, Gschwind et al [50] found no significant differences between the 2 intervention groups or between the SMT group and the control group. This could be due to the SMT program focusing on proprioception, cognitive processing, and balance, which may have shifted the emphasis away from targeting strength. In contrast, the Kinect program may have placed more emphasis on strength-building exercises [50].

The current meta-analysis clearly highlights 2 aspects: the frail and prefrail status of participants and the home-based delivery of VR training. The importance of targeting older adults with frailty or prefrailty originates from the increased vulnerability among this demographic to functional decline and falls, emphasizing the need for tailored rehabilitation interventions [61,62]. Previous systematic reviews and trials examining VR interventions in rehabilitation centers among older populations, rather than specifically individuals with frailty or prefrailty, have reported varied findings. For instance, a recent systematic review [59] showed significant improvements in balance, strength, and mobility among older adults participating in center-based VR programs. Similarly, an RCT [60] delivered in nursing homes showed improvements in balance outcomes, specifically on the BBS, following structured VR interventions. However, such site-specific studies often comprise higher levels of supervision and formal instruction from a therapist, which are not necessarily available in home-based settings. This key distinction highlights the importance of evaluating home-based VR effectiveness independently. Notably, this review found challenges in standardizing intervention intensity and ensuring adherence and safety without direct professional oversight. This is in line with recent findings [63], which reported technology use challenges among older adults without supervision, potentially limiting the effectiveness of home-based VR interventions. Furthermore, studies conducted among older adults often report increased baseline functional ability compared to populations with frailty and prefrailty, possibly overestimating intervention effectiveness. For instance, the study by Donath et al. [64] reported significant improvements in functional performance following a supervised VR training program in healthy older adults. In contrast, our review’s targeted populations with frailty and prefrailty showed smaller effect sizes in mobility outcomes (TUG), possibly due to lower initial functional status.

Overall, our study revealed that VR exercises significantly enhanced balance among older adults compared to those who engaged in regular exercises or remained inactive. However, there were no clear and significant differences in strength and functional mobility. This suggests that while VR training holds potential, its design and implementation require careful consideration to ensure progressive difficulty and proper evaluation through controlled studies.

### Limitations

First, only studies published in English were included, raising the possibility of language bias. This might have caused the exclusion of related studies. While this decision was made to ensure precise analysis of findings, future reviews could compensate for this limitation by using translation resources or multilingual reviewers.

Second, there was considerable heterogeneity in intervention design across the included studies. Variations in VR training, session duration, frequency, and outcome measures made it difficult to directly compare results. The heterogeneity in study protocols could have been a factor in the inconsistent findings. Future studies are advised to design standardized intervention protocols to mitigate these differences.

Third, the small sample sizes in multiple studies limited the statistical power to identify significant outcomes. While we used rigorous quality assessment (eg, PEDro scale) to identify high-quality studies, the small sample size may have resulted in underpowered analyses. Future studies should include larger, adequately powered samples to strengthen the validity of the outcomes.

Fourth, this review excluded gray literature and unpublished studies, which may have introduced publication bias. Although we conducted a comprehensive search across multiple databases to mitigate this risk, future reviews should consider searching clinical trial registries and preprint servers to identify ongoing or unpublished work.

### Future Directions

We should aim to standardize VR protocols. Future studies should develop structured VR interventions with gradual difficulty and standardized outcomes to enhance consistency and comparability across trials.

We should aim to assess long-term effects. Future studies should assess adherence, sustainability of outcomes, and cost-effectiveness over extended periods.

We should aim to enhance engagement and adherence. VR’s interactive nature offers potential benefits beyond physical improvement, including motivation and adherence.

We should aim to explore clinical adoption. Future research should investigate health care providers’ perceptions, barriers, and facilitators to implementing VR-based training in routine geriatric care.

### Conclusion

This systematic review aimed to evaluate the effectiveness of home-based VR training in improving balance, strength, and mobility among older adults with frailty and prefrailty. The findings suggest that VR interventions are consistently effective in improving balance but show limited evidence for improving strength and mobility. These outcomes were influenced by variability in intervention design, duration, and intensity across studies.

## Supplementary material

10.2196/67146 Multimedia Appendix 1 Detailed overview of the search terms and strategies used.

10.2196/67146 Multimedia Appendix 2 Search terms used and description of VR. VR: virtual reality.

10.2196/67146 Checklist 1 PRISMA item checklist. PRISMA: Preferred Reporting Items for Systematic Reviews and Meta-Analyses.

## Abbreviations

BBS: Berg Balance Scale
CS: Chair Stand
MD: mean difference
PEDro: Physiotherapy Evidence Database
PICOS: Population, Intervention, Comparison, Outcome, and Study Design
PRISMA: Preferred Reporting Items for Systematic Reviews and Meta-Analysis
RCT: randomized controlled trial
SMT: Step Mat Training
TUG: Timed Up and Go
VR: virtual reality
