# Supplementary material for: Home-Based Virtual Reality Training for Enhanced Balance, Strength, and Mobility Among Older Adults With Frailty: Systematic Review and Meta-Analysis
Source: JMIR Serious Games. 2025 Jul 18;13:e67146. doi: 10.2196/67146 (PMC12294645; doi:10.2196/67146)
Supplement: Multimedia Appendix 1 — Detailed overview of the search terms and strategies used. [file games-v13-e67146-s001.docx]

# Search terms used

| PubMed database Search Strategy Table | | | | |
| --- | --- | --- | --- | --- |
| Search Number | Query | Filters | Search Details | Results |
| 5 | ''risk of Fall'' OR balance OR strength OR function AND Frail OR Prefrail AND ''Older adult'' AND ''virtual reality'' OR ''Video Games'' OR ''Mobile game'' | Clinical Trial, English, Aged: 65+ years, from 1000/1/1 - 2023/11/1 | Full search string using MeSH terms and field tags | 298 |
| 4 | ''risk of Fall'' OR balance OR strength OR function AND Frail OR Prefrail AND ''Older adult'' AND ''virtual reality'' OR ''Video Games'' OR ''Mobile game'' | Clinical Trial, Aged: 65+ years, from 1000/1/1 - 2023/11/1 | Similar detailed query, slight filter adjustment | 302 |
| 3 | ''risk of Fall'' OR balance OR strength OR function AND Frail OR Prefrail AND ''Older adult'' AND ''virtual reality'' OR ''Video Games'' OR ''Mobile game'' | Clinical Trial, from 1000/1/1 - 2023/11/1 | No language or age restrictions applied | 1,208 |
| 2 | ''risk of Fall'' OR balance OR strength OR function AND Frail OR Prefrail AND ''Older adult'' AND ''virtual reality'' OR ''Video Games'' OR ''Mobile game'' | from 1000/1/1 - 2023/11/1 | Without clinical trial filter | 10,596 |
| 1 | ''risk of Fall'' OR balance OR strength OR function AND Frail OR Prefrail AND ''Older adult'' AND ''virtual reality'' OR ''Video Games'' OR ''Mobile game'' | - | Complete unrestricted search* | 11,934 |

* Search: **''risk of Fall'' OR balance OR strength OR function AND Frail OR Prefrail AND ''Older adult'' AND ''virtual reality'' OR ''Video Games'' OR ''Mobile game''**

(((((("risk"[MeSH Terms] OR "risk"[All Fields] OR "risk of"[All Fields]) AND "fall"[All Fields]) OR ("balance"[All Fields] OR "balanced"[All Fields] OR "balances"[All Fields] OR "balancing"[All Fields]) OR ("strength"[All Fields] OR "strengths"[All Fields]) OR ("functional"[All Fields] OR "functional s"[All Fields] OR "functionalities"[All Fields] OR "functionality"[All Fields] OR "functionalization"[All Fields] OR "functionalizations"[All Fields] OR "functionalize"[All Fields] OR "functionalized"[All Fields] OR "functionalizes"[All Fields] OR "functionalizing"[All Fields] OR "functionally"[All Fields] OR "functionals"[All Fields] OR "functioned"[All Fields] OR "functioning"[All Fields] OR "functionings"[All Fields] OR "functions"[All Fields] OR "physiology"[MeSH Subheading] OR "physiology"[All Fields] OR "function"[All Fields] OR "physiology"[MeSH Terms])) AND ("frail"[All Fields] OR "frails"[All Fields] OR "frailty"[MeSH Terms] OR "frailty"[All Fields] OR "frailness"[All Fields])) OR "Prefrail"[All Fields]) AND ("aged"[MeSH Terms] OR "aged"[All Fields] OR ("older"[All Fields] AND "adult"[All Fields]) OR "older adult"[All Fields]) AND ("virtual reality"[MeSH Terms] OR ("virtual"[All Fields] AND "reality"[All Fields]) OR "virtual reality"[All Fields])) OR ("video games"[MeSH Terms] OR ("video"[All Fields] AND "games"[All Fields]) OR "video games"[All Fields]) OR (("mobile"[All Fields] OR "mobiles"[All Fields]) AND "game"[All Fields])

**Translations**

**''risk of:** "risk"[MeSH Terms] OR "risk"[All Fields] OR "risk of"[All Fields]

**balance:** "balance"[All Fields] OR "balanced"[All Fields] OR "balances"[All Fields] OR "balancing"[All Fields]

**strength:** "strength"[All Fields] OR "strengths"[All Fields]

**function:** "functional"[All Fields] OR "functional's"[All Fields] OR "functionalities"[All Fields] OR "functionality"[All Fields] OR "functionalization"[All Fields] OR "functionalizations"[All Fields] OR "functionalize"[All Fields] OR "functionalized"[All Fields] OR "functionalizes"[All Fields] OR "functionalizing"[All Fields] OR "functionally"[All Fields] OR "functionals"[All Fields] OR "functioned"[All Fields] OR "functioning"[All Fields] OR "functionings"[All Fields] OR "functions"[All Fields] OR "physiology"[Subheading] OR "physiology"[All Fields] OR "function"[All Fields] OR "physiology"[MeSH Terms]

**Frail:** "frail"[All Fields] OR "frails"[All Fields] OR "frailty"[MeSH Terms] OR "frailty"[All Fields] OR "frailness"[All Fields]

**''Older adult'':** "aged"[MeSH Terms] OR "aged"[All Fields] OR ("older"[All Fields] AND "adult"[All Fields]) OR "older adult"[All Fields]

**''virtual reality'':** "virtual reality"[MeSH Terms] OR ("virtual"[All Fields] AND "reality"[All Fields]) OR "virtual reality"[All Fields]

**''Video Games'':** "video games"[MeSH Terms] OR ("video"[All Fields] AND "games"[All Fields]) OR "video games"[All Fields]

**''Mobile:** "mobile"[All Fields] OR "mobiles"[All Fields]

| Scopus database Search Strategy Table | | | |
| --- | --- | --- | --- |
| Search Number | Query | Results |  |
| 5 | ALL(''risk AND of AND fall'' OR balance OR strength OR function AND frail OR prefrail AND ''older AND adult'' AND ''virtual AND reality'' OR ''video AND games'' OR ''mobile AND game'') AND PUBYEAR > 2006 AND PUBYEAR < 2024 AND ( LIMIT-TO ( SUBJAREA,"HEAL" ) OR LIMIT-TO ( SUBJAREA,"MEDI" ) OR LIMIT-TO ( SUBJAREA,"NURS" ) OR LIMIT-TO ( SUBJAREA,"NEUR" ) ) AND ( LIMIT-TO ( DOCTYPE,"ar" ) OR LIMIT-TO ( DOCTYPE,"cp" ) ) AND ( LIMIT-TO ( EXACTKEYWORD,"Human" ) ) AND ( LIMIT-TO ( LANGUAGE,"English" ) ) | 220 |  |
| 4 | ALL(''risk AND of AND fall'' OR balance OR strength OR function AND frail OR prefrail AND ''older AND adult'' AND ''virtual AND reality'' OR ''video AND games'' OR ''mobile AND game'') AND PUBYEAR > 2006 AND PUBYEAR < 2024 AND ( LIMIT-TO ( SUBJAREA,"HEAL" ) OR LIMIT-TO ( SUBJAREA,"MEDI" ) OR LIMIT-TO ( SUBJAREA,"NURS" ) OR LIMIT-TO ( SUBJAREA,"NEUR" ) ) AND ( LIMIT-TO ( DOCTYPE,"ar" ) OR LIMIT-TO ( DOCTYPE,"cp" ) ) | 285 |  |
| 3 | ALL(''risk AND of AND fall'' OR balance OR strength OR function AND frail OR prefrail AND ''older AND adult'' AND ''virtual AND reality'' OR ''video AND games'' OR ''mobile AND game'') AND PUBYEAR > 2006 AND PUBYEAR < 2024 AND ( LIMIT-TO ( SUBJAREA,"HEAL" ) OR LIMIT-TO ( SUBJAREA,"MEDI" ) OR LIMIT-TO ( SUBJAREA,"NURS" ) OR LIMIT-TO ( SUBJAREA,"NEUR" ) ) | 499 |  |
| 2 | ALL(''risk AND of AND fall'' OR balance OR strength OR function AND frail OR prefrail AND ''older AND adult'' AND ''virtual AND reality'' OR ''video AND games'' OR ''mobile AND game'') AND PUBYEAR > 2006 AND PUBYEAR < 2024 | 672 |  |
| 1 | ALL(&apos;&apos;risk AND of AND fall&apos;&apos; OR balance OR strength OR function AND frail OR prefrail AND &apos;&apos;older AND adult&apos;&apos; AND &apos;&apos;virtual AND reality&apos;&apos; OR &apos;&apos;video AND games&apos;&apos; OR &apos;&apos;mobile AND game&apos;&apos;) | **829** |  |

WOS

| Scopus database Search Strategy Table | | | |
| --- | --- | --- | --- |
| Search Number | Query | Results |  |
| 5 | ALL(''risk AND of AND fall'' OR balance OR strength OR function AND frail OR prefrail AND ''older AND adult'' AND ''virtual AND reality'' OR ''video AND games'' OR ''mobile AND game'') AND PUBYEAR > 2006 AND PUBYEAR < 2024 AND ( LIMIT-TO ( SUBJAREA,"HEAL" ) OR LIMIT-TO ( SUBJAREA,"MEDI" ) OR LIMIT-TO ( SUBJAREA,"NURS" ) OR LIMIT-TO ( SUBJAREA,"NEUR" ) ) AND ( LIMIT-TO ( DOCTYPE,"ar" ) OR LIMIT-TO ( DOCTYPE,"cp" ) ) AND ( LIMIT-TO ( EXACTKEYWORD,"Human" ) ) AND ( LIMIT-TO ( LANGUAGE,"English" ) ) | 220 |  |
| 4 | ALL(''risk AND of AND fall'' OR balance OR strength OR function AND frail OR prefrail AND ''older AND adult'' AND ''virtual AND reality'' OR ''video AND games'' OR ''mobile AND game'') AND PUBYEAR > 2006 AND PUBYEAR < 2024 AND ( LIMIT-TO ( SUBJAREA,"HEAL" ) OR LIMIT-TO ( SUBJAREA,"MEDI" ) OR LIMIT-TO ( SUBJAREA,"NURS" ) OR LIMIT-TO ( SUBJAREA,"NEUR" ) ) AND ( LIMIT-TO ( DOCTYPE,"ar" ) OR LIMIT-TO ( DOCTYPE,"cp" ) ) | 285 |  |
| 3 | ALL(''risk AND of AND fall'' OR balance OR strength OR function AND frail OR prefrail AND ''older AND adult'' AND ''virtual AND reality'' OR ''video AND games'' OR ''mobile AND game'') AND PUBYEAR > 2006 AND PUBYEAR < 2024 AND ( LIMIT-TO ( SUBJAREA,"HEAL" ) OR LIMIT-TO ( SUBJAREA,"MEDI" ) OR LIMIT-TO ( SUBJAREA,"NURS" ) OR LIMIT-TO ( SUBJAREA,"NEUR" ) ) | 499 |  |
| 2 | ALL(''risk AND of AND fall'' OR balance OR strength OR function AND frail OR prefrail AND ''older AND adult'' AND ''virtual AND reality'' OR ''video AND games'' OR ''mobile AND game'') AND PUBYEAR > 2006 AND PUBYEAR < 2024 | 672 |  |
| 1 | ALL(&apos;&apos;risk AND of AND fall&apos;&apos; OR balance OR strength OR function AND frail OR prefrail AND &apos;&apos;older AND adult&apos;&apos; AND &apos;&apos;virtual AND reality&apos;&apos; OR &apos;&apos;video AND games&apos;&apos; OR &apos;&apos;mobile AND game&apos;&apos;) | **829** |  |
