# Supplementary material for: Home-Based Virtual Reality Training for Enhanced Balance, Strength, and Mobility Among Older Adults With Frailty: Systematic Review and Meta-Analysis
Source: JMIR Serious Games. 2025 Jul 18;13:e67146. doi: 10.2196/67146 (PMC12294645; doi:10.2196/67146)
Supplement: Multimedia Appendix 2 — Search terms used and description of VR. VR: virtual reality. [file games-v13-e67146-s002.docx]

# **Section S1.** Search terms used.

**Pubmed**

Search ''risk of Fall'' OR balance OR strength OR function AND Frail OR Prefrail AND ''Older adult'' AND ''virtual reality'' OR ''Video Games'' OR ''Mobile game''.

Limited to Clinical Trial and Randomized Controlled Trial.

**WOS**

Search: ''risk of Fall'' OR balance OR strength OR function AND Frail OR Prefrail AND ''Older adult'' AND ''virtual reality'' OR ''Video Games'' OR ''Mobile game'' Filters: Clinical Trial, Humans, English ((((((("risk"[MeSH Terms] OR "risk"[All Fields] OR "risk of"[All Fields]) AND "fall"[All Fields]) OR ("balance"[All Fields] OR "balanced"[All Fields] OR "balances"[All Fields] OR "balancing"[All Fields]) OR ("strength"[All Fields] OR "strengths"[All Fields]) OR ("functional"[All Fields] OR "functional s"[All Fields] OR "functionalities"[All Fields] OR "functionality"[All Fields] OR "functionalization"[All Fields] OR "functionalizations"[All Fields] OR "functionalize"[All Fields] OR "functionalized"[All Fields] OR "functionalizes"[All Fields] OR "functionalizing"[All Fields] OR "functionally"[All Fields] OR "functionals"[All Fields] OR "functioned"[All Fields] OR "functioning"[All Fields] OR "functionings"[All Fields] OR "functions"[All Fields] OR "physiology"[MeSH Subheading] OR "physiology"[All Fields] OR "function"[All Fields] OR "physiology"[MeSH Terms])) AND ("frail"[All Fields] OR "frails"[All Fields] OR "frailty"[MeSH Terms] OR "frailty"[All Fields] OR "frailness"[All Fields])) OR "Prefrail"[All Fields]) AND ("aged"[MeSH Terms] OR "aged"[All Fields] OR ("older"[All Fields] AND "adult"[All Fields]) OR "older adult"[All Fields]) AND ("virtual reality"[MeSH Terms] OR ("virtual"[All Fields] AND "reality"[All Fields]) OR "virtual reality"[All Fields])) OR ("video games"[MeSH Terms] OR ("video"[All Fields] AND "games"[All Fields]) OR "video games"[All Fields]) OR (("mobile"[All Fields] OR "mobiles"[All Fields]) AND "game"[All Fields])) AND ((clinicaltrial[Filter]) AND (humans[Filter]) AND (english[Filter])) Translations ''risk of: "risk"[MeSH Terms] OR "risk"[All Fields] OR "risk of"[All Fields] balance: "balance"[All Fields] OR "balanced"[All Fields] OR "balances"[All Fields] OR "balancing"[All Fields] strength: "strength"[All Fields] OR "strengths"[All Fields] function: "functional"[All Fields] OR "functional's"[All Fields] OR "functionalities"[All Fields] OR "functionality"[All Fields] OR "functionalization"[All Fields] OR "functionalizations"[All Fields] OR "functionalize"[All Fields] OR "functionalized"[All Fields] OR "functionalizes"[All Fields] OR "functionalizing"[All Fields] OR "functionally"[All Fields] OR "functionals"[All Fields] OR "functioned"[All Fields] OR "functioning"[All Fields] OR "functionings"[All Fields] OR "functions"[All Fields] OR "physiology"[Subheading] OR "physiology"[All Fields] OR "function"[All Fields] OR "physiology"[MeSH Terms] Frail: "frail"[All Fields] OR "frails"[All Fields] OR "frailty"[MeSH Terms] OR "frailty"[All Fields] OR "frailness"[All Fields] ''Older adult'': "aged"[MeSH Terms] OR "aged"[All Fields] OR ("older"[All Fields] AND "adult"[All Fields]) OR "older adult"[All Fields] ''virtual reality'': "virtual reality"[MeSH Terms] OR ("virtual"[All Fields] AND "reality"[All Fields]) OR "virtual reality"[All Fields] ''Video Games'': "video games"[MeSH Terms] OR ("video"[All Fields] AND "games"[All Fields]) OR "video games"[All Fields] ''Mobile: "mobile"[All Fields] OR "mobiles"[All Fields].

# **Section S2.** Description of VR used in included studies.

In the current review, several types of VR were identified: (1) Xbox 360 KinectTM consisted of “Kinect Adventures, Kinect Sports, and Kinect Sports Season two” programmes, which included football, tennis, table tennis, skiing, golf, volleyball, and bowling game simulations. (Karahan, Tok et al. 2015)

(2) BTS NIRVANA VR Interactive System; is a VR system developed by BTS Bioengineering which is based on infrared optoelectronic sensors that allow patients to interact with virtual scenes. The system connects to a wall or floor projector and recreates a series of interactive exercises using an infrared camera that analyses the patient's movements (Yeşilyaprak, Yıldırım et al. 2016). Participants were taught to follow the onscreen visual displays and to listen to audio feedback while maintaining their stability during balance activities in a standing posture.

(3) 30-minute video- tape: a videotape shows exercise program which include a 15-minute warm-up focusing on flexibility and dynamic balance exercises, 6 minutes of arm and leg resistance band strengthening exercises, and 5 minutes of aerobic exercise such as walking in place. (Vestergaard, Kronborg et al. 2008)

(4) Tablet PC for exercise administration and feedback, and a necklace-worn motion sensor for daily physical activity registration (Geraedts, Dijkstra et al. 2021)

(5) Computerized step pad system; The step pad is designed so that game is played by stepping in different directions on the pad which challenge speed, accuracy, and balance control. The game requires participants to step as accurately as possible, both in terms of direction and timing, while coinciding their stepping with instructions presented on the screen. For each step feedback was given in the form of a word in the centre of the screen (perfect, good) (Schoene, Lord et al. 2013).

(6) step mat training (SMT) SMT is played using a stepping mat as an input device, requiring participants to repeatedly step in multiple directions under varying cognitive loads, requiring attention, executive control, and rapid processing (Gschwind, Schoene et al. 2015).
